# Study on Secondary Metabolites of Endophytic Fungus, *Aspergillus fumigatus*, from *Crocus sativus* L. Guided byUHPLC-HRMS/MS-Based Molecular Network

**DOI:** 10.1155/2022/7067665

**Published:** 2022-05-09

**Authors:** Yu Jiang, Jing Wu, Hirokazu Kawagishi, Chunxiao Jiang, Qi Zhou, Zheren Tong, Yingpeng Tong, Ping Wang

**Affiliations:** ^1^College of Pharmaceutical Sciences, Zhejiang University of Technology, Hangzhou 310014, China; ^2^Department of Agriculture, Graduate School of Integrated Science and Technology, Shizuoka University, Shizuoka 4228529, Japan; ^3^Institute of Natural Medicine and Health Product, School of Advanced Study, Taizhou University, Taizhou 318000, China

## Abstract

As a traditional Chinese medicine, *Crocus sativus* Linn has been used for a long time in China. However, the studies on secondary metabolites of its endophytic fungi were not fully sufficient. Thus, the endophytic fungus, *Aspergillus fumigatus*, collected from the lateral buds of *C. sativus*, was here investigated. An approach combining UHPLC-HRMS/MS (ultra-high performance liquid chromatography-high resolution mass spectrometry) with molecular network was carried out to construct a molecular network of crude EtOAc extract (CEE) of *A. fumigatus*, in which 32 chemical compounds were annotated. On the basis of analysis results, a total of 15 known natural compounds were isolated from CEE. Among them, compounds 11 and 12 were isolated for the first time from the genus *Aspergillus*. Moreover, CEE and compound 7 exhibited moderate inhibitory activity against *Erwinia* sp. with a MIC value of 100 *μ*g/mL. This study provided a more convenient and rapid approach to investigating the crude extract with complex components of *A. fumigatus*, which is of great benefit to the further study and utilization of secondary metabolites of the genus *Aspergillus*.

## 1. Introduction 

The genus *Aspergillus* is one of the most extensively investigated saprophytic fungal genera [1]. This genus is widely applied in food industries for fermentation, such as sauce making and wine making industries. It is also utilized in processing agricultural products, like biological fertilizers and as a biological control agent. Studies have shown that the genus *Aspergillus* is a rich source of biologically active secondary metabolites such as alkaloids [1, 2], steroids [1, 3], terpenes [4], quinones [5], and polyketides [6], with antimicrobial [1, 7, 8], antitumor [9], antioxidant [10], and anti-inflammatory [11] activities.

UHPLC-HRMS/MS is an important means to identify secondary metabolites of plants and their endophytic fungi [12]. However, this analysis approach will produce a great amount of MS data, the accurate processing of which can be time-consuming and labor-consuming [13]. Since 2014, GNPS (Global Natural Product Society) web platform (http://gnps.ucsd.edu), a data-driven platform for the storage, analysis, and sharing of MS/MS spectra, has been officially open for use. GNPS used with molecular networking is an approach for spectral correlation and visualization that enables the automatic spectral mining of MS data in a few hours [14]. Hence, UHPLC-HRMS/MS-based MN (molecular network), as a method to visualize MS/MS data, can alleviate the above problem of UHPLC-HRMS/MS to a certain degree. It can construct a whole molecular network, formed by numerous nodes and molecular cluster which are grouped and aggregated with structural similarity and MS/MS fragment patterns of compounds [15]. Not only is it used to identify compounds with known structure by comparison with that in the GNPS database, but it also rapidly assigns novel molecules related to known substances in the database to specific structural families, which can accelerate the discovery and characterization process [16, 17].

The dry stigma of *C. sativus* is a precious traditional Chinese medicine with a long history of application, known as “plant gold.” In addition to the medicinal parts of *C. sativus*, its endophytic fungi are also being studied. However, there are just a few of related studies reported, including the field of preparation for secondary metabolites [18–20], community structure and biological characteristics [21], and biological activities [22]. To date, the UHPLC-HRMS/MS analysis of secondary metabolites of the genus *Aspergillus* of endophytic fungi collected from *C. sativus* has not been reported.

In our current work, UHPLC-HRMS/MS-based MN approach, a fast and effective method, was utilized to investigate CEE of *A. fumigatus*, the endophytic fungus from *C. sativus*, constructing a molecular network and identifying 30 chemical components. Using the annotated molecular network as a guide, we carried out further isolation. A total of 15 known natural compounds were isolated, namely, eight alkaloids, two anthraquinones, two benzoate derivatives, one long chain unsaturated fatty acid ester, and two terpenoids. Additionally, several isolated compounds and CEE were evaluated for their antibacterial activities against plant pathogenic bacteria. This work supplied a more rapid and effective approach to investigating the crude extract with complex components of *A. fumigatus*, which is very beneficial for the further study and utilization of secondary metabolites of the genus *Aspergillus*.

## 2. Materials and Methods

### 2.1. Chemicals and Materials

Chromatogram grade and LC-MS grade MeOH and MeCN were purchased from Shanghai Macklin Biochemical Co., Ltd. (Shanghai, China) and Fisher (Waltham, USA), respectively. Analytically pure reagents, including EtOH, EtOAc, formic acid, MeOH, acetone, CH_2_Cl_2_, petroleum ether (PE), *n*-BuOH, and CHCl_3_, and chemically pure NaCl were all obtained from Shanghai Zhanyun Chemical Co., Ltd. (Shanghai, China). Streptomycin with USP grade (Sangon Biotech Co., Ltd., Shanghai, China) was used as the positive control for antibacterial experiment.

The fungal strain, *A. fumigatus*, was isolated from lateral buds of *C. sativus* at the Jiande Sandu Saffron Professional Cooperative, Zhejiang Province, on May 7^th^, 2019. The strain was deposited at Taizhou University under the GenBank accession No. MZ854147.

### 2.2. Fermentation, Extraction, and Isolation

Fermented solid medium (120 g rice, 150 mL ultrapure water in 1 L Erlenmeyer flask, 140 flasks, 21 days) was soaked with EtOAc five times at room temperature. The crude extracts (CE, 182.1 g) were obtained with subsequent merging and concentration. Then, after suspension in water and extraction with PE, EtOAc, and *n*-butanol in turn, the layers of EtOAc were combined and concentrated under vacuum to prepare CEE (49.3 g). Furthermore, CEE was dissolved in MeOH and filtered for further UHPLC-Q-TOF-MS (ultra-high performance liquid chromatography tandem quadrupole time-of-flight mass spectrometry) analysis.

The CEE was subjected to silica gel column chromatography (CC) and then eluted with a gradient solvent system of CH_2_Cl_2_-EtOAc (1 : 0 to 1 : 1, v/v) and CH_2_Cl_2_–MeOH (5 : 1 to 0 : 1, v/v) to harvest eleven fractions (Fr. E1 to E11).

Fr. E3 (2.73 g) was divided on silica gel CC (PE-EtOAc = 50 : 1 to 2 : 1, v/v), and thirteen fractions (Fr. E3.1 to E3.13) were collected. Fr. E3.5 (130.1 mg) was separated via preparative TLC twice (PE-EtOAc = 1 : 1 and CH_2_Cl_2_-EtOAc = 3 : 1, v/v, respectively) to yield compound 1 (5.6 mg). Fr. E3.11 (1.12 g) was purified by silica gel CC eluted with CH_2_Cl_2_–MeOH (1 : 0 to 100 : 1, v/v) and then was chromatographed on Sephadex LH-20 CC (CH_2_Cl_2_– MeOH = 1 : 1, v/v) to get compounds 2 (72.4 mg) and 3 (27.6 mg).

Fr. E4 (4.59 g) was precipitated to obtain compound 4 (1.35 g). The filtrate after removing 4 was applied to Sephadex LH-20 CC and eluted with CH_2_Cl_2_–MeOH (1 : 1, v/v) to yield seven fractions (Fr. E4.1 to Fr. E4.7). Fr. E4.3 (34.0 mg) was purified by preparative TLC (CH_2_Cl_2_– MeOH = 50 : 1, v/v) to give compound 5 (8.9 mg). In a similar way, compound 7 (39.0 mg) was also obtained using preparative TLC (PE-EA = 1 : 4, v/v) from Fr. E4.7. Fr. E4.4 was subjected to semipreparative HPLC (MeCN–H_2_O = 60 : 40, v/v) to yield compound 6 (1.8 mg, *t*_*R*_ = 12.4 min).

Fr. E6 (2.16 g) was chromatographed on silica gel and eluted using PE-EA (2 : 1 to 0 : 1) to obtain thirteen fractions (Fr. E6.1 to Fr. E6.13). Fr. E6.4 (394 mg) was purified via Sephadex LH-20 CC (CH_2_Cl_2_–MeOH = 1 : 1, v/v) and preparative TLC (CH_2_Cl_2_/acetone = 3 : 1) to obtain compound 12 (11.7 mg). Fr. E6.6 (575.6 mg) was loaded on Sephadex LH-20 CC (CH_2_Cl_2_–MeOH = 1 : 1, v/v) to yield five fractions (Fr. E6.6.1 to E6.6.5). Fr. E6.6.4 was separated over silica gel CC (CH_2_Cl_2_–MeOH = 1 : 0 to 2 : 1) to give nine fractions (Fr. E6.6.4.1 to E6.6.4.9). Compounds 13 (2.5 mg, *t*_R_ = 26.2 min) [HPLC mobile phase: MeCN–H_2_O = 80 : 20, v/v] and 8 (50.9 mg, *t*_*R*_ = 12.4 min) [HPLC mobile phase: MeCN–H_2_O = 65–35, v/v] were obtained by semipreparative HPLC from Fr. E6.6.4.2 and Fr. E6.6.4.5, respectively. Fr. E6.6.4.4 was separated by preparative TLC (PE/acetone = 1 : 1, v/v) to get compound 14 (3.4 mg). Compound 11 (4.1 mg, *t*_*R*_ = 16.9 min) was given via semipreparative HPLC (MeCN–H_2_O = 35 : 65, v/v) from Fr. E6.6.5. Fr. E6.7 (118.1 mg) was subjected to Sephadex LH-20 CC (CH_2_Cl_2_–MeOH = 1 : 1, v/v) and further purified by semipreparative HPLC (MeCN–H_2_O = 41 : 59, v/v) to give compound 9 (5.5 mg, *t*_*R*_ = 29.7 min). Fr. E6.9 (181.3 mg) was separated using Sephadex LH-20 CC (CH_2_Cl_2_–MeOH = 1 : 1, v/v) to obtain seven fractions (Fr. E6.9.1 to E6.9.7). Compounds 10 (2.9 mg, *t*_*R*_ = 15.0 min) and 15 (1.1 mg, *t*_*R*_ = 17.9 min) were yielded by semipreparative HPLC (MeCN–H_2_O = 41 : 59, v/v) from Fr. E6.9.4.

### 2.3. UHPLC-HRMS/MS Conditions

UHPLC-HRMS/MS was performed with an Exactive™ MS (Thermo Scientific, Sunnyvale, CA, USA) equipped with HESI-II, and an Ultimate R3000 UHPLC (Thermo Fisher Scientific) with an ACQUITY UPLC HSS T3 column (1.8 *μ*m, 2.1 × 100 mm, Waters Corporation, Milford, CT, USA). The measurement temperature was maintained at 30°C with flow rate of 0.3 mL/min, injection volume of 5 *μ*L, and DAD detection wavelength of 254 nm. The mobile phase was MeCN (solvent A) and 0.5% formic acid-water solution (solvent B), and the elution condition was as follows: 0–10 min, 5% A; 10–20 min, 5–40% A; 20–45 min, 40–90% A; 45–50 min, 90% A; 50–50.01 min, 90–5% A; 50.01–57 min, 5% A.

Ionization source and scanning mode of mass spectrometer were electrospray ion (ESI) sources and negative ion detection mode, respectively. The mass spectrometry conditions were as follows: scanning range, m/z 100–1500; spray voltage, −3.0 kV; sheath gas pressure, 40 arb; auxiliary gas pressure, 10 arb; capillary temperature, 350°C; heater temperature, 350.

### 2.4. Data Analysis with UHPLC-HRMS/MS-Based MN Approach

The MS/MS data analysis was conducted with data processing by GNPS and the construction of MN, and the detailed process was as follows. The GNPS_Vendor_Conversion software downloaded from GNPS web platform was used to convert the format of MS/MS data from RAW to mzXML. Subsequently, the data with mzXML format were imported to MZmine 2.5.3 for data preprocessing, in which the parameters were modified by Tong et al. [13]. Then, the processing data were uploaded on GNPS web platform and analyzed based on the Feature-Based Molecular Networking (FBMN). All MS/MS fragments within the range of m/z 17 of precursor were removed for data filtering. Only the top six ion fragments in the 50 Da window were selected for MS/MS window filter. The precursor and MS/MS fragment ion mass tolerance were both set to 0.075 Da. After the basic options, the cosine score of filtering edge was higher than 0.7, and matched fragment ions were more than 5. Meanwhile, the matched score threshold of the network spectra and library spectra was kept higher than 0.7, and there were at least 5 library search matched peaks. Finally, the data were exported via the link http://gnps.ucsd.edu/ProteoSAFe/status.jsp?task=ae5bf0640bdf48138c97edacfae4cbf7 and visualized using Cytoscape 3.8.2 software to construct the MN.

### 2.5. Preparation of Standard and Sample Solutions

The standard stock solutions of the two compounds, questin (4) and 12,13-dihydroxyfumitremorgin C (8), were solved in MeOH with concentrations of 500 *μ*g/mL and 60 *μ*g/mL, respectively. 2 mg of CEE was solved with 1 mL MeOH. The standard and sample solutions were filtered through a polyvinylidene difluoride (PVDF) filter of 0.45 *μ*m and kept at 4°C for analysis.

### 2.6. Method Validation

#### 2.6.1. Calibration Curve and Sensitivity

Calibration curves of questin and cyclotryprostatin A were calculated based on the peak areas (Y) and concentrations of standard solutions (X). The limit of detection (LOD) and limit of quantification (LOQ) for each compound had a signal-to-noise ratio (S/N) of 3 and 10, respectively.

#### 2.6.2. Precision, Stability, and Recovery

The precision was investigated by a sample solution at one concentration level in six replicates with variations expressed by relative standard deviations (RSD). The stability was tested with one of the sample solutions, which was kept at 4°C in the refrigerator and taken out for analysis at 0, 1, 2, 4, and 8 h. The recovery was assessed by spiking analytes into the sample to evaluate the accuracy of method.

### 2.7. Bioassay

The microbroth dilution method was used to evaluate antibacterial activities against four plant pathogenic bacteria (*Agrobacterium tumefaciens*, *Pantoea agglomerans*, *Ralstonia solanacearum*, and *Erwinia* sp., provided by Ningbo testobio Co., Ltd., Zhejiang, China) on 96-well culture plates [23]. Streptomycin was used as positive control at initial concentration of 200 *μ*g/mL, diluted with 4% DMSO solution. The tested bacteria were incubated in a thermostatic oscillator (30°C, 150 rpm) for 12 h with NA broth (1 g yeast extract, 3 g beef exact, 5 g peptone, 5 g glucose, and 1 g agar in 1 L medium, adjusting pH to 7.2 with NaOH) to get bacterial suspension. After adjusting the bacterial concentration to 1 × 10^5^–1 × 10^6^ CFU/mL with NA broth, the bacterial dilution was poured into 96-well culture plates with 50 *μ*L per hole. The inception solutions (compounds 7, 13, and 15 with concentration of 200 *μ*g/mL and CEE with concentration of 400 *μ*g/mL) with 50 *μ*L were added to the first hole and mixed evenly. 50 *μ*L of solutions in the first hole was drawn with a pipette gun to be transferred to the second hole and mixed well. The operation was repeated until the twelfth hole according to the double dilution method in triplicate. MIC (minimal inhibitory concentrations) was determined after incubation at 30°C for 24 h.

## 3. Results and Discussion

### 3.1. Identification of Secondary Metabolites in CEE by UHPLC-HRMS/MS-Based MN

**Figure 1:**
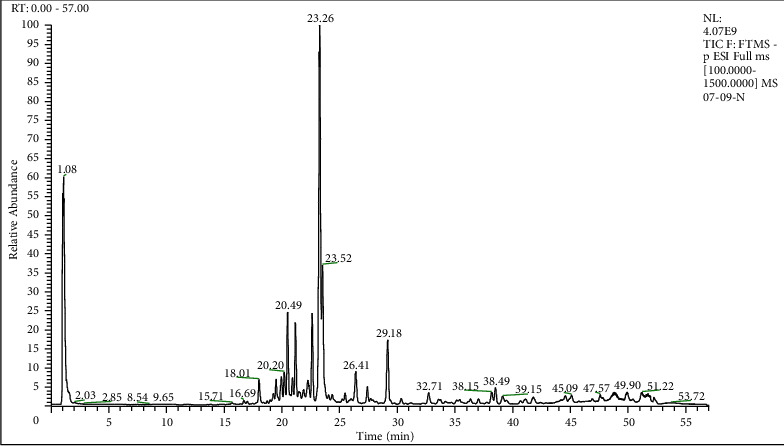
The total ion chromatogram of CEE analyzed by UHPLC-HRMS/MS in negative ion mode.

**Figure 2:**
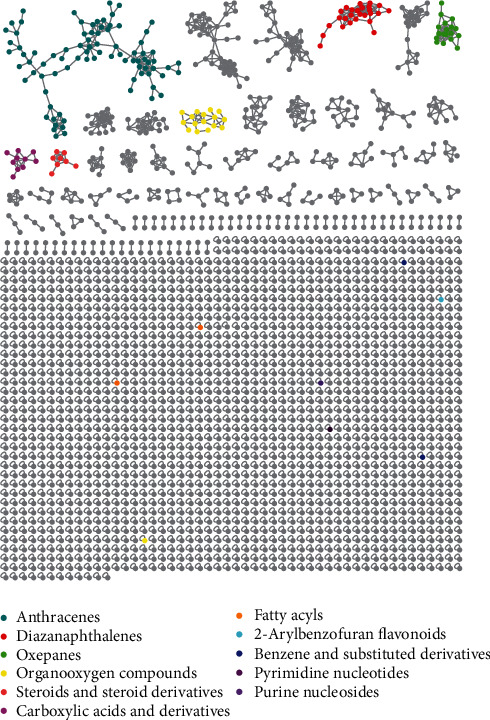
The entire MS/MS molecular network obtained from CEE.

The CEE was analyzed by UHPLC-HRMS/MS (Figure 1), and the data were uploaded to GNPS web platform to establish molecular network with annotation of GNPS. As illustrated in Figure 2, 2387 precursor ions were organized into a molecular network with 110 clusters and 1766 nodes. Different structure types of compounds were identified in the GNPS database from the MN, including 2-arylbenzofuran flavonoids, anthracenes, benzene and substituted derivatives, carboxylic acids and derivatives, diazanaphthalenes, fatty acyls, organooxygen compounds, and pyrimidine nucleotides. In the UHPLC-HRMS/MS-based MN, 32 nodes of CEE were annotated (Table 1). Among them, 8 compounds—namely, four anthraquinones, emodin [24], physcion [25], carviolin [26], and endocrocin [27]; two alkaloids, pseurotin A [28] and fumiquinazoline C [29]; and two benzoate derivatives, methyl asterrate [30] and asterric acid [31]—have been reported as the secondary metabolites of the genus *Aspergillus*.

**Figure 3:**
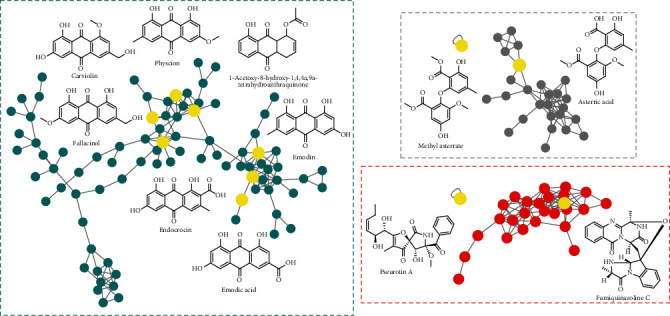
Subnetwork of tandem MS/MS molecular working for CEE.

Compounds with similar structure are grouped into the same molecular cluster in molecular network because of some identical ion fragments, which was also verified in literature [32, 33]. As shown in Figure 3, the above-mentioned four anthraquinones and the other three annotated anthraquinones—1-acetoxy-8-hydroxy-1,4,4a,9a-tetrahydroanthraquinone; emodic acid; and fallacinol—were clustered into the same molecular subnetwork, which matched the above law. However, this law cannot apply to all compounds, such as alkaloids and benzene derivatives. The two identified alkaloids and benzoate derivatives were found to be nodes in different clusters (Figure 3). In the meantime, it could be considered that it also contained other anthraquinones, alkaloids, and benzene derivatives with similar structure in CEE. Thus, the subsequent separation was carried out based on the analysis results.

### 3.2. Isolation of Secondary Metabolites in CEE-Based GNPS-MN

**Figure 4:**
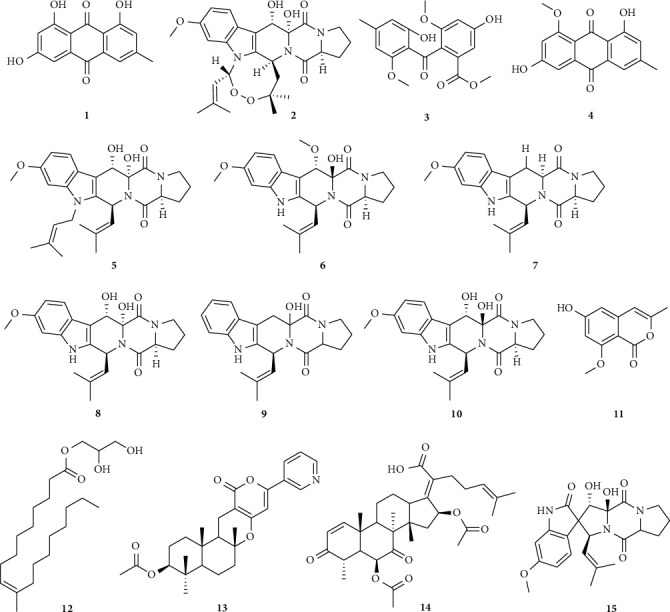
Chemical structure of compounds 1–15.

On the basis of GNPS-MN results, 15 known compounds were isolated, and their structures are described in Figure 4. Through comparison of the NMR spectroscopic data with that reported in the literature, the known compounds were identified as emodin (1) [34], verruculogen (2) [35], monomethylsulochrin (3) [36], questin (4) [34], fumitremorgins B–C (5, 7) [37], cyclotryprostatins A-B (10, 6) [38], 10-methyl-9Z-octadecenoic glyceride (12) [39], pyripyropene E (13) [40], helvolic acid (14) [41], 12,13-dihydroxyfumitremorgin C (8) [35], 6-hydroxy-8-methoxy-3-methylisocoumarin (11) [42], 13-dehydroxycyclotryprostatin C (9) [43], and spirotryprostatin A (15) [44]. Notably, compounds 11 and 12 have not been isolated from the genus *Aspergillus*.

**Figure 5:**
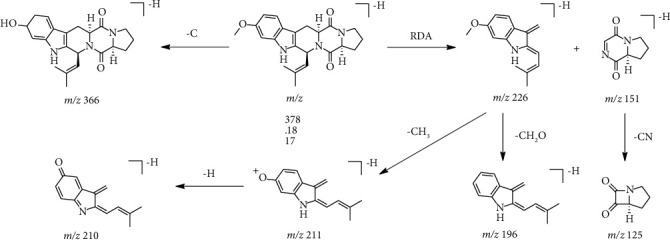
Proposed fragmentation pathways of [M-H]^−^ ions for compound 7 observed in CEE.

The isolated compounds were also identified by combination of UHPLC-HRMS/MS with GNPS-MN, shown in Table 2. Among them, there were 7 structurally similar indole alkaloids (compounds 2, 5, 6, 7, 8, 9, and 10), featuring consistent 6/5/6/6/5 heteropentacyclic ring core, and compound 7 was taken as an example to elaborate the mass spectral fragmentation pathways of alkaloids with this structure (Figure 5). Obviously, compound 7 was extremely prone to Retro-Diels–Alder (RDA) fragmentation [45] to form characteristic ions m/z 226 [M-H-C_7_H_8_N_2_O_2_]^−^ and 151 [M-H-C_15_H_17_NO]^−^. Additionally, under collision voltage of mass spectrum, the compound formed a more stable structure through various successive dissociation processes, including decarbonization (m/z 366 [M-H-C]^−^), demethylation (m/z 211 [M-H–C_7_H_8_N_2_O_2_–CH_3_]^−^), dehydrogenation (m/z 210 [M-H–C_7_H_8_N_2_O_2_–CH_3_–H]^−^), dealdehyding (m/z 196 [M-H–C_7_H_8_N_2_O_2_–HCHO]^−^), and decyanation (m/z 125 [M-H–C_15_H_17_NO–CN]^−^). Compounds 2, 5, 6, 8, 9, and 10 possessed similar fragmentation pathways to those of compound 7, especially RDA fragmentation, and were identified by MS/MS data and GNPS-MN.

However, these alkaloids were not clustered into the same molecular subnetwork but distributed in several single nodes. According to judgement, the reason for this situation lies in the various substituent groups of different compounds. It might form characteristic ions with diverse mass-to-charge ratio, which could not be analyzed and integrated by GNPS platform to be grouped into the same clusters. Meanwhile, the alkaloids with this type of structure would also possess other dissociation processes randomly, like decarbonylation, dehydration, and deamination, leading the m/z differences between compounds. These were also the reasons why the above compounds with structure of indole alkaloids were distributed in single nodes rather than clustered into other subnetworks.

### 3.3. Method Validation

The characteristics of calibration curves of each standard compound, including regression equation, correlation coefficient, LOD, and LOQ, are shown in Table 3. The high correlation coefficient values (*R*^2^ ≥ 0.9997) displayed good linearity over a relatively wide range of concentration. In the precision test, RSDs were less than 1.37%, a result which indicated that the precision met the acceptability criteria for sample analysis. In terms of stability, RSDs were 0.63% and 1.78%, respectively, showing that analytes did not degrade significantly with storage of sample solution at 4 for 8 h. The RSDs of recovery test were less than 2.55%, which demonstrated the reliability and accuracy of the measurement of these compounds. These results, with an acceptable range of values, are listed in Table 4.

### 3.4. Antibacterial Assay

The compounds 7, 13, and 15 and CEE were evaluated for their antibacterial activities against four plant pathogenic bacteria (*Agrobacterium tumefaciens*, *Pantoea agglomerans*, *Ralstonia solanacearum*, and *Erwinia* sp.) through the microbroth dilution method in 96-well culture plates. Compound 7 and CEE both showed selective and moderate inhibitory activity against *Erwinia* sp. (MIC = 100 *μ*g/mL). However, compounds 13 and 15 were devoid of antibacterial activity against the four plant pathogenic bacteria (Table 5). *Erwinia* sp., as a Gram-negative bacterium, is usually parasitic on plants and can cause rot to infringe on plants owing to its own pectin polygalacturonase. Thus, it could be considered that compound 7 and CEE might be used for inhibition of Gram-negative bacterial, and prevention and treatment of plant diseases caused by Gram-negative bacterial to some extent.

## 4. Conclusion

In the present investigation, uncovered by UHPLC-HRMS/MS-based MN strategy, 30 nodes were annotated from CEE of *A. fumigatus*, the endophytic fungus from the lateral buds of *C. sativus*. Meanwhile, 15 compounds were isolated according to the analysis results. Among them, CEE and compound 7 showed moderate inhibitory effect with a MIC value of 100 *μ*g/mL against the plant pathogenic bacteria, *Erwinia* sp. This study provided a more rapid and convenient means to investigate the crude extract of *A. fumigatus*, which is greatly beneficial to the further study and utilization of secondary metabolites of the genus *Aspergillus* and even other plants and fungi.

## Figures and Tables

**Table 1:** Characterization of compounds in CEE using UHPLC-HRMS/MS-based MN.

No.	Identification	Formula	*T*_*R*_ (min)	[M-H]^−^	HPLC-MS^2^ m/z (% base peak)
1	Emodin	C_15_H_10_O_5_	26.32	269.0454	269.0454 (100), 241.0502 (21.99), 225.0553 (51.87), 210.0314 (5.21), 197.0602 (8.59), 185.0602 (2.81), 182.0367 (3.99)
2	4-Acetamido-butyric acid	C_6_H_11_NO_3_	1.67	144.0655	144.0655 (26.5), 126.0549 (11.51), 102.0549 (100), 100.0757 (74.66), 98.06 (4.83), 94.2577 (1.64), 84.0443 (5.01), 58.0287 (51.4)
3	1,6-Anhydro-*β*-glucose	C_6_H_10_O_5_	0.98	161.0446	113.0232 (20.42), 101.0233 (46.48), 97.0283 (12.26), 88.0395 (22.03), 85.0283 (70.62), 73.0283 (53.24), 71.0127 (100), 59.0127 (59.43)
4	Sorbitol	C_6_H_14_O_6_	0.95	181.0712	181.0712 (21.97), 163.0607 (7.24), 119.034 (3.69), 101.0233 (40.79), 89.0232 (33.69), 85.0284 (14.03), 73.0284 (17.64), 71.0127 (100), 59.0127 (84.07)
5	Citric acid	C_6_H_8_O_7_	1.05	191.0189	191.0189 (3.95), 129.0187 (4.21), 112.011 (6.64), 111.0077 (100), 87.0077 (73.19), 85.0284 (41.43), 67.0178 (9.26), 59.0125 (2.05), 57.0335 (12.1)
6	D-Gluconic acid	C_6_H_12_O_7_	1.00	195.0504	195.0504 (29.87), 177.0397 (1.19), 159.0292 (1.43), 129.0183 (20.29), 99.0076 (8.11), 87.0075 (13.41), 75.0076 (100), 72.9919 (13.19), 59.0127 (27.37)
7	Diethyl phthalate	C_12_H_14_O_4_	22.27	221.0820	221.082 (17.12), 198.4325 (11.63), 177.8034 (11.7), 134.0368 (11.6), 121.0284 (47.49), 118.212 (11.44), 75.0229 (16.99), 71.0492 (100), 69.0334 (49.48)
8	N-acetyltryptophan	C_13_H_14_N_2_O_3_	16.79	245.0926	245.0926 (8.2), 203.0818 (34.28), 159.0925 (6.75), 142.0653 (12.2), 130.0657 (6.52), 116.0495 (47.93), 98.0236 (30.13), 74.0236 (100), 58.0287 (37.6)
9	Mannose 6-phosphate	C_6_H_13_O_9_P	1.19	259.0126	259.0126 (15.03), 198.9911 (4.26), 171.0056 (14.96), 138.9698 (8.21), 128.0343 (40.97), 96.959 (100), 78.9579 (79.78)
10	Inosine	C_10_H_12_N_4_O_5_	1.22	267.0728	267.0728 (10.87), 135.0303 (100), 126.0301 (7.53), 113.0234 (6.4), 92.0243 (9.06), 89.0232 (18.26), 71.0127 (21.61), 59.0127 (63.06)
11	Dibutyl phthalate	C_16_H_22_O_4_	29.18	277.1441	206.0638 (14.43), 147.0078 (21.16), 134.0361 (69.7), 127.1118 (100), 121.0285 (76.05), 75.023 (18.48), 72.0943 (15.59)
12	Physcion	C_16_H_12_O_5_	30.82	283.0609	283.0609 (20.38), 268.0379 (1.16), 241.0458 (14.84), 240.0424 (100), 212.048 (3.88), 184.052 (1.22)
13	N-fructosyl pyroglutamate	C_11_H_17_NO_8_	1.04	290.0873	290.0873 (3.57), 200.0561 (4.44), 170.0453 (1.64), 168.0659 (1.61), 128.0343 (100), 84.0443 (3.87)
14	(10E,12Z)-9-oxooctadeca-10,12-dienoic acid	C_18_H_30_O_3_	24.39	293.2119	236.1055 (25.64), 221.1541 (100), 220.1465 (74.18), 205.1234 (13.8), 192.1158 (10.1), 177.0918 (9.11), 161.7506 (8.59), 125.1129 (7.9), 81.1714 (6.98)
15	1-Acetoxy-8-hydroxy-1,4,4a,9a-tetrahydroanthraquinone	C_16_H_14_O_5_	23.31	285.0676	285.0676 (2.14), 284.0641 (13.09), 283.0609 (26.16), 268.0375 (1.26), 241.0457 (39), 240.0424 (100), 212.0475 (2.55), 184.0521 (0.85)
16	5-Hydroxy-6,4′-dimethoxy-isoflavone	C_17_H_14_O_5_	24.74	297.0767	297.0775 (28.43), 256.0381 (22.34), 255.0615 (19.11), 254.0582 (100), 239.0349 (17.13)
17	Emodic acid	C_15_H_8_O_7_	21.77	299.0192	299.0192 (100), 255.0292 (39.56), 227.0343 (30.72), 211.0395 (58.04), 199.0393 (6.15), 183.0447 (22.38), 167.0493 (15.54)
18	4,6-Dihydroxy-2-[(3-hydroxy-4-methoxyphenyl)methylene]-3(2-H)-benzofuran-one	C_16_H_12_O_6_	22.57	299.0557	299.0567 (14.27), 285.0357 (16.97), 284.0325 (100), 257.0413 (14.56), 256.0375 (81.62), 240.1302 (2.15), 228.0425 (14.26), 209.4983 (2.15), 200.0475 (22.96), 199.0398 (8.03)
19	Carviolin	C_16_H_12_O_6_	20.95	299.0560	299.056 (27.75), 257.0416 (14.59), 256.0375 (100), 255.0293 (3.42), 228.0425 (3.86), 227.0348 (1.81), 211.0406 (1.58), 200.0477 (3.43)
20	Fallacinol	C_16_H_12_O_6_	20.77	299.0563	299.0563 (38.6), 285.0368 (6.4), 284.0319 (22.56), 257.0402 (17.84), 256.0375 (100), 253.1716 (4.05)
21	Juniperoside III	C_15_H_20_O_7_	32.63	311.1686	311.1686 (91.15), 216.0092 (20.99), 197.0269 (2.54), 184.0192 (28.75), 183.0114 (100), 113.9287 (2.81), 104.8775 (2.63), 96.9588 (4.1), 79.9563 (4.81),
22	Endocrocin	C_16_H_10_O_7_	20.47	313.0352	313.0352 (21.37), 270.0492 (18.39), 269.0453 (100), 242.0544 (3.75), 241.0502 (19.76), 226.0586 (9.33), 225.0553 (62.29), 197.06 (9.25), 181.0653 (6.96)
23	Avocadyne acetate	C_19_H_34_O_4_	35.48	325.1839	325.1839 (77.39), 216.0091 (14.12), 197.0273 (3.77), 185.007 (4.55), 184.0188 (29.07), 183.0114 (100), 119.0483 (2.66), 79.9561 (2.65)
24	Canrenone	C_22_H_28_O_3_	39.10	339.1995	339.1995 (98.55), 239.0736 (1.53), 197.0269 (3.91), 185.0062 (2.36), 184.0187 (26.4), 183.0114 (100), 163.112 (45.89), 119.0491 (2.3)
25	Asterric acid	C_17_H_16_O_8_	22.81	347.0768	271.0616 (14.8), 257.0411 (13.81), 256.0374 (100), 228.0429 (21.66), 212.0476 (43.06), 181.05 (71.39), 166.0262 (94.78), 149.0235 (65.61), 122.0363 (54.5), 105.0335 (95.57)
26	Methyl asterrate	C_18_H_18_O_8_	22.88	361.2017	329.067 (75.53), 270.0531 (50.27), 254.0584 (40.55), 240.0419 (39.44), 227.0349 (70.34), 225.0554 (100), 211.0395 (99.88), 195.0447 (25.93), 183.0445 (58.24), 105.0336 (14.61)
27	8–5′-Benzofuran-diferulic acid	C_20_H_18_O_8_	17.53	385.1233	341.1033 (9.58), 326.0802 (6.77), 311.0574 (8.64), 297.1147 (9.5), 282.0891 (14.46), 267.066 (100), 266.0558 (15.04), 249.0552 (8.47), 239.0708 (35.64), 221.0607 (11.56), 211.076 (14.45), 193.065 (7.29)
28	Pseurotin A	C_22_H_25_NO_8_	19.51	430.1505	200.0353 (78.02), 188.0347 (47.53), 160.0397 (57.23), 148.0397 (39.72), 139.0391 (90.8), 125.0235 (79.89), 111.044 (84.11), 97.0283 (77.7), 83.049 (100)
29	Fumiquinazoline C	C_24_H_21_N_5_O_4_	21.13	442.1526	442.1526 (13.66), 240.0774 (23.48), 225.0539 (16.53), 212.0822 (6.37), 199.0508 (13.59), 188.0827 (5.48), 170.0354 (6.69), 156.0448 (3.76), 145.0398 (100), 132.0446 (5.37)
30	Obassioside B	C_25_H_28_O_11_	0.96	503.1339	383.1191 (13.98), 221.0666 (18.35), 161.0449 (13.32), 119.0339 (19.78), 113.0233 (28.91), 101.0234 (59.98), 97.0284 (9.99), 89.0233 (64.87), 85.0284 (24.94), 73.0283 (26.31), 71.0127 (72.42), 59.0127 (100)
31	N-Acetyl-phenylalanine	C_11_H_13_NO_3_	15.68	206.0815	206.0815 (18.39), 165.0741 (10.58), 164.071 (100), 147.0442 (78.21), 118.9923 (4.26), 91.0541 (30.96), 72.008 (23.03), 70.0286 (13.57), 58.0287 (88.22)
32	Arabinofuranosyluracil	C_9_H_12_N_2_O_6_	1.35	243.0617	243.0629 (4.77), 200.0556 (7.45), 152.0348 (10.53), 122.0238 (16.76), 111.0264 (7.23), 110.0236 (100), 94.0285 (6.86), 82.0287 (68.21), 66.0337 (22.67)

**Table 2:** Characterization of isolated compounds from CEE.

No.	Identification	Formula	*T*_*R*_ (min)	[M-H]^−^	HPLC-MS^2^ m/z (% base peak)
1	Emodin	C_15_H_10_O_5_	26.32	269.0454	269.0454 (100), 241.0502 (21.99), 225.0553 (51.87), 210.0314 (5.21), 197.0602 (8.59), 185.0602 (2.81), 182.0367 (3.99)

2	Verruculogen	C_27_H_33_N_3_O_7_	28.09	510.2240	469.2944 (16.17), 451.2852 (100), 339.2335 (23.34), 255.1754 (13.42), 137.0963 (11.04), 121.065 (56.28), 83.0491 (60.42)

3	Monomethylsulochrin	C_18_H_18_O_7_	23.51	345.0977	331.546 (1.14), 313.0715 (4.03), 267.0286 (1.2), 254.0576 (4.36), 225.0549 (2.4), 211.0402 (2.36), 181.0499 (100), 166.0263 (89.93), 138.0312 (18.23), 123.0079 (7.27), 122.0364 (16.7), 95.0127 (6.33)

4	Questin	C_16_H_12_O_5_	20.90	283.0597	283.0597 (16.53), 270.0549 (5.81), 241.0456 (14.95), 240.0424 (100), 227.0347 (19.7), 221.7869 (2.85), 211.0397 (8.68)

5	Fumitremorgin B	C_27_H_33_N_3_O_5_	25.51	478.2342	460.2263 (34.71), 293.142 (33.93), 280.1706 (17.87), 265.1461 (42.99), 264.1396 (37.74), 196.0758 (29.76), 179.0455 (54.39), 153.0662 (83.03), 125.0347 (100)

6	Cyclotryprostatin B	C_23_H_27_N_3_O_5_	20.76	424.1885	424.1885 (37.65), 393.0933 (29.63), 366.1812 (31.15), 228.0411 (96.88), 212.355 (32.23), 211.0989 (46.84), 210.0917 (75.84), 185.0362 (36.82), 167.0453 (60.78), 154.433 (31.58), 139.0505 (100), 111.0191 (28.43)

7	Fumitremorgin C	C_22_H_25_N_3_O_3_	22.16	378.1817	366.0103 (13.84), 226.1229 (19.52), 211.0991 (57.72), 210.0918 (100), 196.0764 (35.07), 125.0345 (22.89)

8	12,13-Dihydroxyfumitremorgin C	C_22_H_25_N_3_O_5_	18.29	410.1717	320.3534 (15), 308.1407 (28.5), 303.6974 (15), 294.1198 (19), 293.1173 (100), 245.3081 (16), 227.0945 (30.5), 194.1525 (13.5), 156.9944 (14), 139.0505 (44), 128.6233 (17.5), 109.9676 (13)

9	13-Dehydroxycyclotryprostatin C	C_21_H_23_N_3_O_3_	21.64	364.1665	301.1026 (18.05), 245.0099 (15.29), 231.4112 (14.83), 209.0329 (15.1), 196.113 (50.94), 180.081 (100), 167.0453 (34.14), 128.9646 (12.37), 123.8069 (16.17),

10	Cyclotryprostatin A	C_22_H_25_N_3_O_5_	18.29	410.1717	320.3534 (15), 308.1407 (28.5), 303.6974 (15), 294.1198 (19), 293.1173 (100), 245.3081 (16), 227.0945 (30.5), 194.1525 (13.5), 156.9944 (14), 139.0505 (44), 128.6233 (17.5), 109.9676 (13)

11	6-Hydroxy-8-methoxy-3-methylisocoumarin	C_11_H_10_O_4_	16.32	205.0500	205.05 (100), 190.0272 (8.45), 162.9824 (13.4), 149.0237 (24.47), 148.0522 (30.42), 118.9923 (24.54), 105.0335 (13.48), 75.0021 (13.39), 63.7466 (5.22)

12	10-Methyl-9*Z*-octadecenoic glyceride	C_22_H_42_O_4_	39.77	369.3005	369.3005 (32.67), 351.2911 (17.38), 308.3032 (27.04), 307.3002 (100), 124.6359 (11.64), 98.5349 (11.38), 87.0824 (9.88), 72.9919 (11.66)

14	Helvolic acid	C_33_H_44_O_8_	29.52	567.2959	527.2982 (21.61), 509.2892 (38.37), 483.3128 (20.4), 405.2802 (100), 321.2231 (10.58), 217.1237 (28.98), 199.148 (17.03), 161.0599 (15.26), 135.0806 (47.45), 121.065 (32.57)

15	Spirotryprostatin A	C_22_H_25_N_3_O_6_	21.60	426.1680	426.1680 (29.29), 270.1132 (32.67), 255.0894 (76.24), 225.0796 (60.94), 210.0559 (100), 196.0764 (34.1), 167.0457 (35.77), 154.0504 (21.46), 139.0507 (26.44), 112.0395 (41.68)

**Table 3:** Linear regression data, LOD, and LOQ of standard compounds.

Analyte	Regression equation	*R* ^2^	Linear range (*μ*g/mL)	LOD (*μ*g/mL)	LOQ (*μ*g/mL)
Questin	*Y* = 26.534*X* − 116.56	0.9997	6.25–500.00	0.15	0.56
Cyclotryprostatin A	*Y* = 689.23*X* − 9.3319	1	0.60–24.00	0.13	0.59

**Table 4:** Analytical results of precision, stability, and recovery tests.

Analyte	Precision (RSD %)	Stability RSD (%)	Recovery RSD (%)
Questin	1.37	0.63	2.55
Cyclotryprostatin A	0.59	1.78	1.25

**Table 5:** Antibacterial activity data of compounds 7, 13, and 15 and CEE.

Samples	MIC (*μ*g/mL)			
	*A. tumefaciens*	*P. agglomerans*	*R. solanacearum*	*Erwinia* sp.
7	>100	>100	>100	100
13	>100	>100	>100	>100
15	>100	>100	>100	>100
CEE	>100	>100	>100	100
Streptomycin	100	50	50	25

## Data Availability

The data used to support the findings of this study are available from the corresponding author upon request.

## Supplementary Materials

Supplementary Materials The NMR spectra for isolated compounds (compounds 1–15) are available in the Supplementary Materials document.
