# Supplementary material for: Study on Secondary Metabolites of Endophytic Fungus, Aspergillus fumigatus, from Crocus sativus L. Guided byUHPLC-HRMS/MS-Based Molecular Network
Source: Int J Anal Chem. 2022 May 9;2022:7067665. doi: 10.1155/2022/7067665 (PMC9110225; doi:10.1155/2022/7067665)
Supplement: Supplementary Materials — The NMR spectra for isolated compounds (compounds 1–15) are available in the Supplementary Materials document. [file 7067665.f1.docx]

**Supplementary File**

1. **Supplementary Figures**

*Figure S.1 (a):* *^1^H-NMR (400 MHz, DMSO-d_6_) spectrum of compound* ***1*** *(emodin)*

*Figure S.1 (b): ^13^C-NMR (100 MHz, DMSO-d_6_) spectrum of compound* ***1*** *(emodin)*

*Figure S.2 (a): ^1^H-NMR (400 MHz, CDCl_3_) spectrum of compound* ***2*** *(verruculogen)*

*Figure S.2 (b): ^13^C-NMR (400 MHz, CDCl_3_) spectrum of compound* ***2*** *(verruculogen)*

*Figure S.3 (a): ^1^H-NMR (400 MHz, CDCl_3_) spectrum of compound* ***3*** *(monomethylsulochrin)*

*Figure S.3 (b): ^13^C-NMR (400 MHz, CDCl_3_) spectrum of compound* ***3*** *(monomethylsulochrin)*

*Figure S.4 (a):* *^1^H-NMR (400 MHz, DMSO-d_6_) spectrum of compound* ***4*** *(questin)*

*Figure S.4 (b): ^13^C-NMR (100 MHz, DMSO-d_6_) spectrum of compound* ***4*** *(questin)*

*Figure S.5 (a): ^1^H-NMR (400 MHz, CDCl_3_) spectrum of compound* ***5*** *(Fumitremorgin B)*

*Figure S.5 (b): ^13^C-NMR (400 MHz, CDCl_3_) spectrum of compound* ***5*** *(Fumitremorgin B)*

*Figure S.6 (a): ^1^H-NMR (400 MHz, CDCl_3_) spectrum of compound* ***6*** *(Cyclotryprostatin B)*

*Figure S.6 (b): ^13^C-NMR (400 MHz, CDCl_3_) spectrum of compound* ***6*** *(Cyclotryprostatin B)*

*Figure S.7 (a): ^1^H-NMR (400 MHz, CDCl_3_) spectrum of compound* ***7*** *(Fumitremorgin C)*

*Figure S.7 (b): ^13^C-NMR (400 MHz, CDCl_3_) spectrum of compound* ***7*** *(Fumitremorgin C)*

*Figure S.8 (a): ^1^H-NMR (400 MHz, CDCl_3_) spectrum of compound* ***8*** *(10-methyl-9Z-octadecenoic glyceride)*

*Figure S.8 (b): ^13^C-NMR (400 MHz, CDCl_3_) spectrum of compound* ***8*** *(10-methyl-9Z-octadecenoic glyceride)*

*Figure S.9 (a): ^1^H-NMR (400 MHz, CDCl_3_) spectrum of compound* ***9*** *(pyripyropene E)*

*Figure S.9 (b): ^13^C-NMR (400 MHz, CDCl_3_) spectrum of compound* ***9*** *(pyripyropene E)*

*Figure S.10 (a): ^1^H-NMR (400 MHz, CDCl_3_) spectrum of compound* ***10*** *(helvolic acid)*

*Figure S.10 (b): ^13^C-NMR (400 MHz, CDCl_3_) spectrum of compound* ***10*** *(helvolic acid)*

*Figure S.11 (a): ^1^H-NMR (400 MHz, CDCl_3_) spectrum of compound* ***11*** *(12,13-dihydroxyfumitremorgin C)*

*Figure S.11 (b): ^13^C-NMR (400 MHz, CDCl_3_) spectrum of compound* ***11*** *(12,13-dihydroxyfumitremorgin C)*

*Figure S.12 (a): ^1^H-NMR (400 MHz, (CD_3_)_2_CO) spectrum of compound* ***12*** *(6-hydroxy-8-methoxy-3-methylisocoumarin)*

*Figure S.12 (b): ^13^C-NMR (400 MHz, (CD_3_)_2_CO) spectrum of compound* ***12*** *(6-hydroxy-8-methoxy-3-methylisocoumarin)*

*Figure S.13 (a): ^1^H-NMR (400 MHz, (CD_3_)_2_CO) spectrum of compound* ***13*** *(13-dehydroxycyclotryprostatin C)*

*Figure S.13 (b): ^13^C-NMR (400 MHz, (CD_3_)_2_CO) spectrum of compound* ***13*** *(13-dehydroxycyclotryprostatin C)*

*Figure S.14(a): ^1^H-NMR (400 MHz, (CD_3_)_2_CO) spectrum of compound* ***14*** *(cyclotryprostatin A)*

*Figure S.14 (b): ^13^C-NMR (400 MHz, (CD_3_)_2_CO) spectrum of compound* ***14*** *(cyclotryprostatin A)*

*Figure S.15(a): ^1^H-NMR (400 MHz, (CD_3_)_2_CO) spectrum of compound* ***15*** *(spirotryprostatin A)*

*Figure S.15 (b): ^13^C-NMR (400 MHz, (CD_3_)_2_CO) spectrum of compound* ***15*** *(spirotryprostatin A)*
